# Circularly polarized luminescence as a contingency assay for ‘mirror life’ bio-hazards

**DOI:** 10.1098/rsos.260921

**Published:** 2026-10-21

**Authors:** Lewis E. MacKenzie, Dominic J. Black, Robert Pal

**Affiliations:** ^1^ Department of Pure and Applied Chemistry, University of Strathclyde, Glasgow, UK; ^2^ Department of Chemistry, Durham University, Durham, UK

**Keywords:** chirality, circularly polarized luminescence, stereochemistry, origin of life, mirror life, mirror bacteria, biosecurity, bio-surveillance, existential risk

## Abstract

Mirror life presents a low-likelihood but high-severity global biosecurity threat that has attracted the attention of bioscientists, governments and international security organizations. However, current analytical techniques are not fit to identify mirror life biohazards. Herein, we propose the first assay method for screening against mirror life. This approach is based upon optical circularly polarized luminesce (CPL) signatures generated by fluorescent probes, which bind to subcellular targets (e.g. membranes, organelles, nucleic acids, etc.). Probes can either be achiral or be intrinsically chiral. We set out why a multi-probe reporter panel approach incorporating many CPL-generating probes is necessary to identify mirror life with a high degree of confidence (i.e. avoiding false positives). The distinct CPL signatures can be analysed via recent innovations in CPL detection, i.e. rapid CPL spectroscopy, CPL microscopy (enantioselective differential chiral contrast, EDCC, imaging) and CPL EDCC cameras. While these are advanced technologies, they share the same fundamental optical principles as three-dimensional cinema glasses and so are amenable to scaling and portable applications. The CPL assay approach to mirror life screening could be a useful tool for screening against mirror life biohazards and ensuring compliance with any potential future international legislation against mirror-life research.

## 1. The risks of mirror life

Life as we know it is predominantly of a single chirality set: the 20 encoded amino acids are predominantly left-handed—although some minor abundance of mirror forms are extant—whereas sugars and nucleic acid forms are predominantly right-handed ( table 1) [1,12,13]. At larger scales, chirality also manifests in cells and organisms [14]. Why ‘life as we know it’ has developed with conventional terrestrial chirality remains unclear and is a topic of ongoing and intense scientific research and debate [2,13,15–18]. ‘Mirror life’ is a speculative form of life, composed of the opposite chirality set to conventional terrestrial life. Mirror life could either be of natural origin or human-made. Given the pace of advancements in synthetic biology and synthetic cells [19], the hazards of artificial mirror life are being pre-emptively considered by both bioscientists and governments.

**Table 1.** Chirality of some prominent biological molecules/proteins/structures.*

biological molecules/proteins/structures	conventional terrestrial chirality	hypothesized mirror life chirality
**nucleic acids, i.e. DNA and RNA**	right-handed [1–3]	left-handed
**Z-DNA**	left-handed [4,5]	right-handed
**encoded amino acids**	left-handed [2]	right-handed
**alpha helices**	right-handed [6]	left-handed
**beta-sheets**	right-handed [7]	left-handed
**actin filaments**	right-handed [8]	left-handed
**sugars**	right-handed [2]	left-handed
**collagen triple helix**	right-handed [9]	left-handed
**amyloid plaques**	right-handed [10]	left-handed
**phospholipids in membrane proteins—bacteria**	right-handed [11]	left-handed
**phospholipids in membrane proteins—eucharia**	right-handed [11]	left-handed
**phospholipids in membrane proteins—archaea**	left-handed [11]	right-handed

* N.B. this is not exhaustive and opposite chirality forms of various molecules/proteins/structures can occur naturally at lower abundance.

It should be noted that there are highly speculative scenarios for encountering extant mirror life, such as the discovery of isolated reservoirs of speculated primordial terrestrial mirror life [20,21] or extraterrestrial mirror life, e.g. encountered via comet, meteorite or exploration of other planets (figure 1B) [22]. These are generally regarded as low-likelihood compared with artificial mirror life. Therefore, discussion is generally focused on artificial mirror life biohazards.

**Figure 1.**
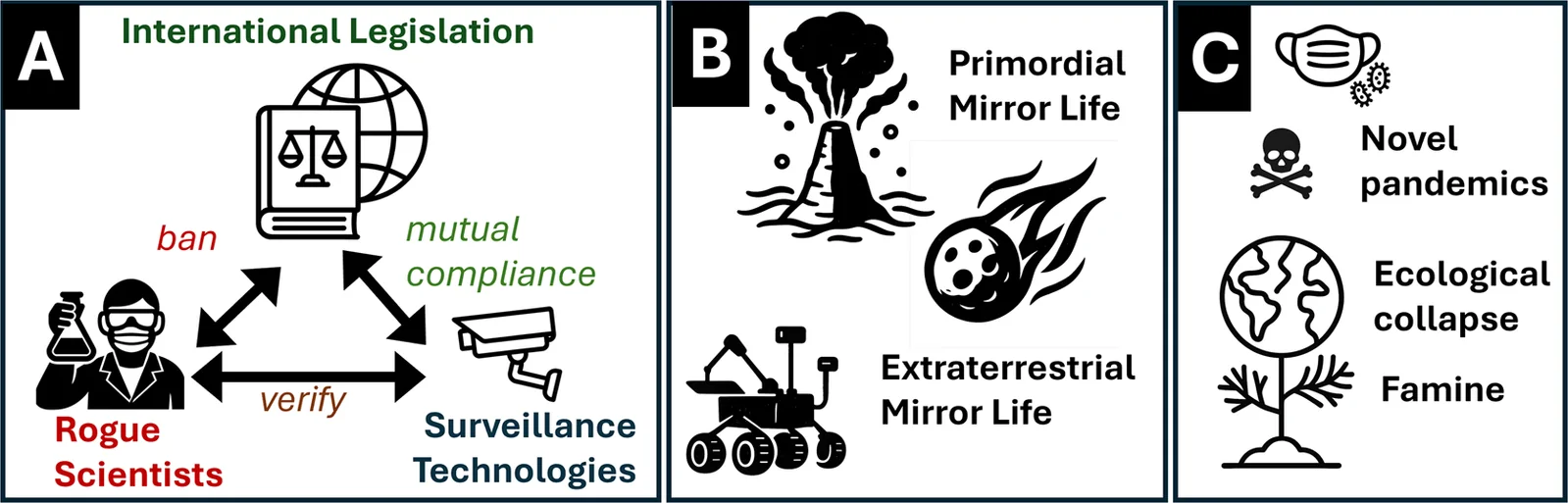
Summary of mirror life control measures and risks. (A) Contingency surveillance technologies and international legislation may be required to prevent artificial mirror life research. (B) Some speculative scenarios where mirror life could be encountered. (C) Existential risks to Earth's ecosphere if mirror life is encountered and not contained.

In a landmark December 2024 policy perspective, Adamala *et al.* [23] provided a comprehensive justification for why artificial mirror life—if allowed to develop—could pose an existential threat to life on Earth. Adamala *et al.* [23] concluded that the most likely route to mirror life hazards in the near future would be artificially produced mirror bacteria. Such mirror life bacteria could invade Earth's ecosystems and evade immune systems, potentially resulting in lethal infections in humans, animals and plants (i.e. irrevocable global harm). Further, Adamala *et al.* [23] noted that countermeasures against mirror bacteria, such as mirror antibiotics, mirror phages, etc., are unlikely to be able to control mirror bacteria. Therefore, the hazards of mirror bacteria to life on Earth are severe, presenting an existential risk to humanity (figure 1A,C). Consequently, Adamala *et al.* [23] have proposed the need for pre-emptive international biosecurity legislative controls to minimize the risk of artificial mirror life arising (i.e. banning mirror life research owing to the severe hazards and minimal scientific benefit [23]).

In 2025 and 2026, the hazards of mirror life have found wider attention from scientists, governments and international bodies. Adamala's recommendations were endorsed by Germany's Central Commission for Biological Safety (ZKBS) in September 2025 [24]. In January 2025, the United States of America (USA) Congressional Research Service published an ‘in focus’ article by Kuiken, examining considerations around mirror life and discussing the prior calls for mirror life research moratoriums [25]. The UNESCO International Bioethics Committee also mentioned the risks of mirror life in a 2025 report [26]. There have been several scientific meetings focused on mirror life hazards: in May 2025 there was the Carnegie Endowment for International Peace Workshop on International Governance of Mirror Life, in June 2025 the Paris Conference on Risks from Mirror Life, and in September 2025 the Manchester Technical Workshop on Mirror Life [27,28]. In January 2025, the UK Government Office for Science convened an expert meeting on the risks of mirror life, which was chaired by the UK's Chief Scientific Advisor [29]. The records of the meeting clearly set out the need for new analytical methods for screening against mirror life. Of particular relevance are the following notes from that meeting:

— Note 23. ‘We won't understand many risks associated with mirror life unless it comes into being’ [29].
— Note 24. ‘Current biosurveillance methods to detect threats to health would not be able to detect mirror life because they only recognize natural chirality’ [29].
— Note 28. ‘There should be a coalition among funders, researchers, governments and civil society to develop appropriate guidelines to manage the development of mirror molecules and prevent the development of replicating mirror organisms’ [29].
— Note 29. ‘There should be collective international agreement to monitor research into self-replicating mirror cells and to develop appropriate mitigations on a case-by-case basis. Any such agreement could, of course, be ignored by bad actors’ [29].

Mirror life continues to draw scrutiny in 2026. In January 2026, the Science and Security Board Bulletin of Atomic Scientists identified mirror life as an existential biological risk as a factor in their ‘doomsday clock’ calculations [30]. In February 2026, the China Arms Control and Disarmament Association (China) and Nuclear Threat Initiative (USA) published a joint statement on Shared Priorities for Strengthened Biosecurity & Responsible AI-Biotechnology Innovation calling for international frameworks to guard against mirror life [31]. Most recently, the United Nations Secretary-General's Scientific Advisory Board published a briefing on mirror life in March 2026 [32].

There is therefore an imperative need for analytical techniques and assay technologies that could plausibly detect mirror life hazards if they arise. Herein, we set out the first known approach for detecting and screening against mirror life biohazards by exploiting circularly polarized luminescence (CPL) emitted from both chiral and achiral fluorophores. The proposed mirror life assay approach could be developed safely with testing on conventional terrestrial life, and the necessary analytical equipment can be deployed in portable formats to identify and contain mirror life biohazards *in situ* if they ever arise.

### 1.1. General principle for a mirror life assay based upon circularly polarized luminescence

Herein we set out a ‘road map’ for all-optical assay techniques for detecting mirror life based upon CPL. In physics, circular polarization of photons corresponds to photon spin angular momentum [33–35], but in everyday life, CPL is best known as the type of light used in stereoscopic three-dimensional display technologies [36]. It is well established that CPL can be generated from various classes of chiral light-emitting molecules (figure 2) [40] or via achiral light-emitting molecules arranged into supramolecular chiral systems (§1.2; figure 3A). Such light-emitting CPL-active systems emit a unique CPL signature, which can then be detected and quantified via spectroscopy, imaging and microscopy [38,41,42]. Further, suitably designed chiral molecules will selectively bind to subcellular targets (i.e. different organelles), with CPL emission carrying information about the chiral binding state and chiral configuration of the CPL emitter probe [38,39]. This is further discussed for achiral probes (§1.2), chiral probes (§1.3); read-out considerations (§2). Assuming that mirror life is chemically analogous to conventional terrestrial life, these CPL signals could, in principle, be used to assess chirality and thereby provide a method for screening against mirror life.

**Figure 2.**
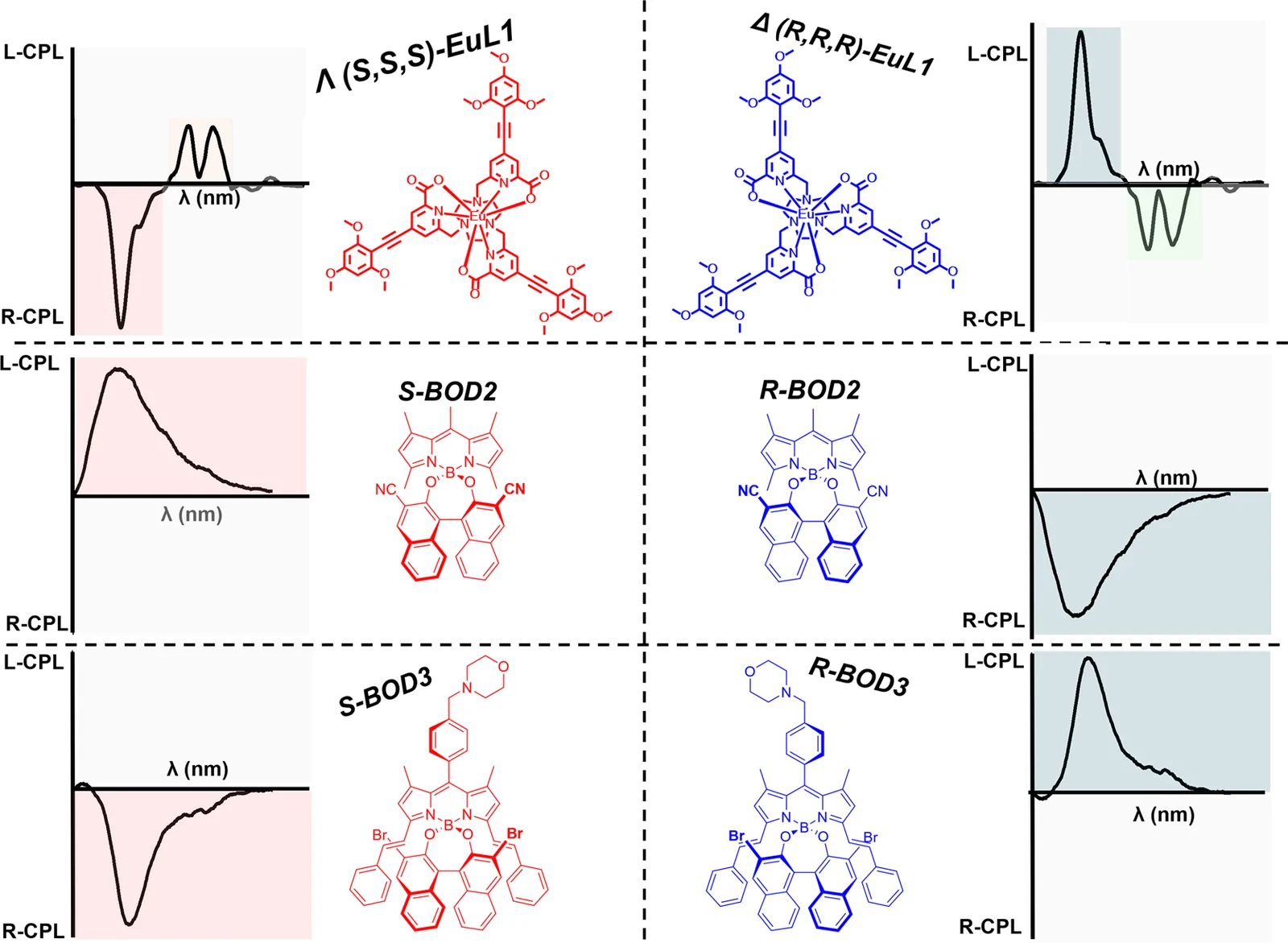
Three selected examples of enantiomers of CPL-active chiral luminescent/fluorescent compounds and their representative CPL spectra. Top row: a red-emitting chiral europium complex, EuL1 [CPB up to 345 M^–1^ cm^–1^ @ 594 nm in MeCN], which has been used in CPL enantioselective differential chiral contrast (EDCC) imaging for security inks [37], with a parent complex in which enantioselectivity localizes to mitochondria (Λ enantiomer) and liposomes (Δ enantiomer) in live NIH 3T3 cells [38]. Middle row: BOD2, a green-emitting chiral BODIPY of CPB = 4 M^–1^ cm^–1^ @ 529 nm in CHCl3. Bottom row: BOD3, a red-emitting chirally-locked bis-perylene diimide macrocycle of CPB = 34 M^–1^ cm^–1^ @658 nm in CHCl3. BOD2 and BOD3 exhibit enantioselective binding to the lysosome in live NIH 3T3 cells [39]. Red and blue shaded areas on each CPL spectrum indicate the wavelength range and CPL polarization sign for analysis and imaging of each enantiomer.

**Figure 3.**
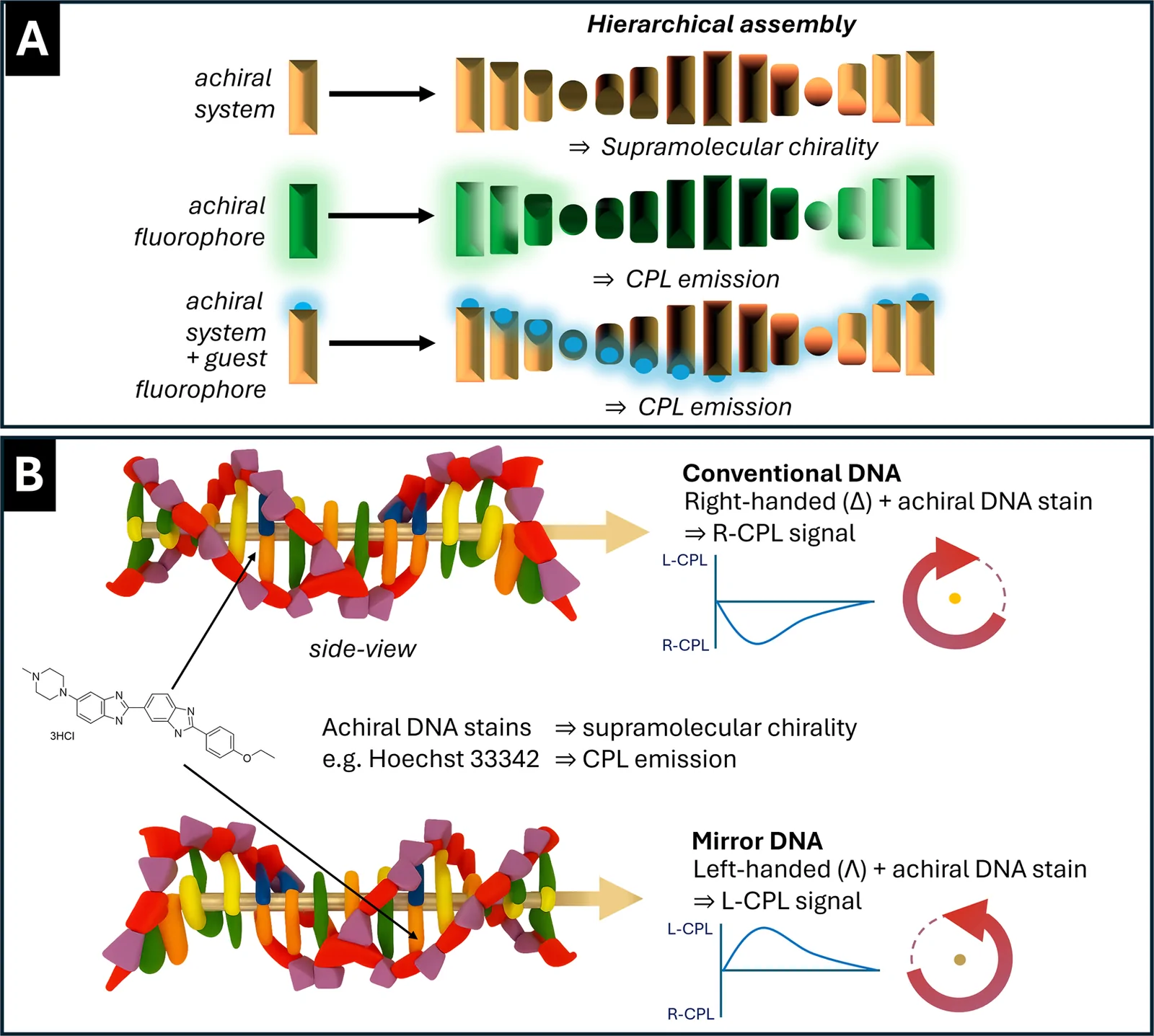
Supramolecular chirality and its application to DNA labelling for mirror life detection. (A) Simplified general example of how supramolecular chirality can arise from achiral subunits and generate CPL. The achiral subunits can either be intrinsically fluorescent or be a guest fluorophore. (B) An example of how achiral fluorophores, e.g. the commonly used DNA stain, Hoechst 33342, could be used to bind to either conventional DNA or mirror DNA, thereby generating a distinct CPL signature which could be used to discriminate against mirror life.

### 1.2. Mirror life assay based upon supramolecular chirality of achiral fluorophores

The most general way CPL could be used for mirror life discrimination is to use existing achiral fluorescent/luminescent probes, which when bound to chiral biological structures, generate supramolecular chirality (figure 3). Supramolecular chirality is a well-known process that has been demonstrated many times with systems, such as achiral fluorophores and nanoparticles [43–52]. The most obvious target for screening against mirror life with achiral fluorescent probes is the helical structure of DNA (figure 3B) [1], which is right-handed in conventional terrestrial life and which would be left-handed for mirror life. Common achiral DNA-binders, such as DAPI [53] or Hoechst dyes [54] (minor groove binders), could be co-opted to achieve CPL via supramolecular effects. In general, a wide range of mirror groove DNA binders are available and offer advanced functionalities, such as serving as Raman reporters or antimicrobial agents [55,56]. Even water is known to form chiral structures when bound to the minor groove of DNA [57]. Intercalating fluorophores (e.g. YoYo family dyes) may also serve to generate CPL via supramolecular chirality, but their interactions with DNA must be carefully considered [58–60]. Non-canonical forms of DNA, including quad-stranded G-quadruplexes, may give rise to unusual CPL generation effects owing to their unique structure [61]. Additionally, fluorescent probes for RNA may be considered as potential CPL generators, with due consideration for diverse and sophisticated behaviours and functions of RNA [62–69]. Of course, there are plenty of other helical biological structures which could also serve the purposes of inducing CPL via supramolecular chirality; these are noted in table 1. Indeed, amino acids are known to induce CPL when bound to otherwise racemic lanthanide complexes [70]. A full consideration of how achiral fluorescent probes may interact with RNA and other chiral biomolecules to generate CPL is beyond the scope of this perspective. However, an elegant experimental test of our approach would be to bind and analyse CPL generated by achiral fluorophores to both DNA (right-handed) and natural left-handed Z-DNA (not mirror DNA) [4,5]; this would provide a biosecure means of validating our approach without invoking mirror-life biohazards. Figure 4 provides an illustrative example of how DNA binding achiral dyes would be read out in a mirror life assay via CPL microscopy.

**Figure 4.**
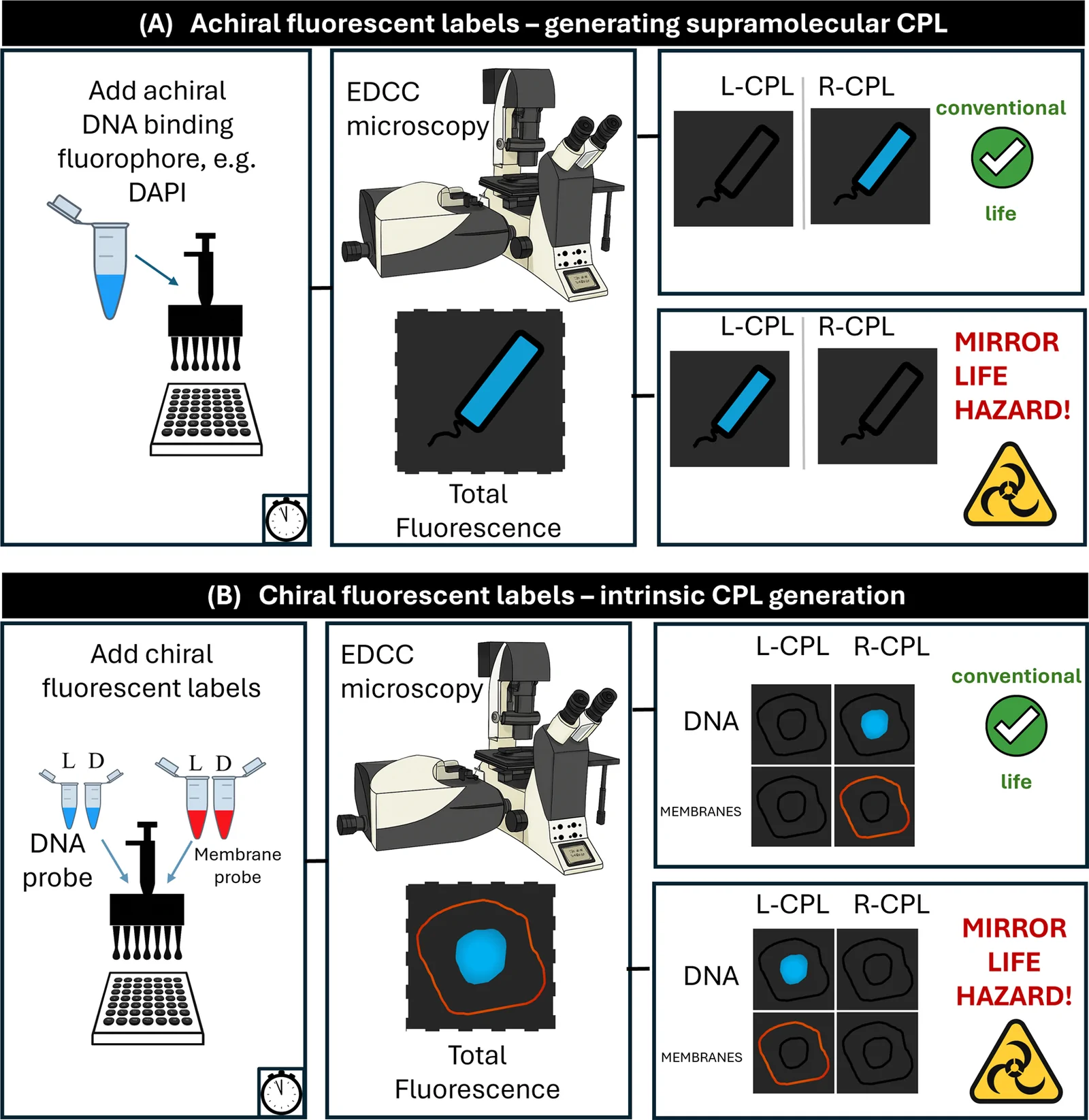
Depiction of how CPL EDCC microscopy imaging could be used as a read-out for a mirror life assay. (A) Using achiral fluorophores, e.g. an achiral DNA binder (blue) to identify DNA chirality in bacteria. (B) Using chiral CPL-generating probes, which bind to, for example, DNA (blue) and membranes (red) to identify chirality signatures of mammalian cells.

### 1.3. Assay against mirror life with enantiomers of chiral light-emitting compounds

In recent decades, a wide array of CPL-active molecules have been reported [40], with some being designed to probe cells and biological molecules [38,39]. Usually for a de novo chemical system, a pair of enantiomers is synthesized, and the subcellular binding/localization of each enantiomer is studied. [38,71] Often, despite being chemically identical, different enantiomers will localized to different subcellular features owing to enantioselective interactions [38,39,72,73]. This is also true for chiral nanoparticles, where left- and right-handed nanoparticles exhibit different uptake properties [74–78]. Thus, using chemically identical enantiomers of a CPL-active chiral probe could provide a basis for identifying/distinguishing anomalous mirror life. In theory, any enantiopure chiral molecular probe engineered to specifically label conventional terrestrial life could be used to discriminate against terrestrial analogue mirror life *apriori*. Figure 4 provides an illustrative example of how such CPL-active chiral probes would be read out in a mirror life assay via CPL microscopy.

### 1.4. Potential false positives and the need for robust verified assay panels

Any assay may have false positives, and our proposed method is no exception: rational design and careful testing of mirror twin enantiomer probes are required to avoid false positives for mirror life. For example, bacteria and eukaryotic cells have membranes comprising primarily *R*-phospholipids, whereas archaea have membranes comprised mainly of *S*-phospholipids (table 1) [11]. Therefore, targeting these membrane phospholipids alone would be a recipe for false positives in mirror life detection! Likewise, often both L- and D-enantiomers of amino acids, sugars and biomolecules naturally exist in terrestrial life, just that one enantiomer is in abundance [22]. One example is left-handed Z-DNA, which is found *in vivo* during transcription [79]. Therefore, to avoid false positives, a robust verified multi-reporter panel of CPL-active mirror-life probes would be required, with the appropriate CPL-signature and intensity for each probe having to match that anticpated from mirror life.

## 2. Technologies for read out of circularly polarized luminescence-based mirror life assays

Owing to recent technological advancements circa 2016–2025, rapid CPL detection technologies (i.e. spectroscopy, imaging and microscopy) are now available and could be used for the detection of mirror life [38,42,80]. Indeed, the optical principles behind CPL detection are fundamentally the same as the technology used in low-cost, mass-produced three-dimensional cinema glasses [81]. The only difference is the quality of the optical components involved. The current technology readiness levels (TRLs) of various CPL detection/read-out technologies at time of writing are summarized in table 2.

**Table 2.** Technology readiness level for circularly polarized luminescence detection/read-out technologies.

CPL detection technology	estimated TRL
**rapid CPL spectrometers**	4 (validated in laboratory environment)
**CPL EDCC cameras**	4 (validated in laboratory environment)
**CPL EDCC confocal microscope**	4 (validated in laboratory environment)
**CPL plate readers**	2 (technology concept and/or application formulated)

*For NASA definitions of TRL, see [82].

TRL estimations are correct at the time of writing.

CPL microscopy, also known as enantioselective differential chiral contrast (EDCC) imaging, can simultaneously discriminate between L- and D-enantiomers of luminescent probe molecules inside single living cells at confocal resolution (i.e. approx. 126 nm lateral resolution, 396 nm axial resolution) [38]. Extension to rapid spectroscopic detection [80] could also facilitate the analysis of multiple probes at once, i.e. for panel detection. In conjunction with suitable CPL-active probes, EDCC microscopy would therefore be able to distinguish even a single mirror-life cell among conventional life (generic examples in figure 4). Therefore, we expect that CPL EDCC microscopy would be best used for testing of high-priority targets, such as suspect meteorites (extraterrestrial mirror life hazard) or laboratory swabs. Such optical detection technologies will be compatible with existing biosecurity/biocontainment procedures involving imaging/microscopy. The ability of CPL EDCC microscopy to discriminate a single mirror life bacterium would eliminate the risks associated with the need to culture/grow suspect specimensin bulk, thereby contributing to reducing mirror life contamination hazards.

In 2023, the first EDCC CPL-based camera system was reported; the design adapted a commercial polarization-sensitive camera with a solid-state quarter-wave plate to analyse and measure CPL signals (figure 5) [42]. Such EDCC-CPL cameras could be made in a handheld format and could be particularly useful for rapid *in situ* screening against mirror life hazards, i.e. inspection of door handles or other potentially contaminated surfaces. Further, camera-style sensors are widely used in space systems, interplanetary probes and planetary rovers, so EDCC imaging in such environments beyond Earth is plausible [83–86]. However, the effects of turbidity on CPL signals (e.g. depolarization) should be considered for remote sampling applications in complex turbid environs.

**Figure 5.**
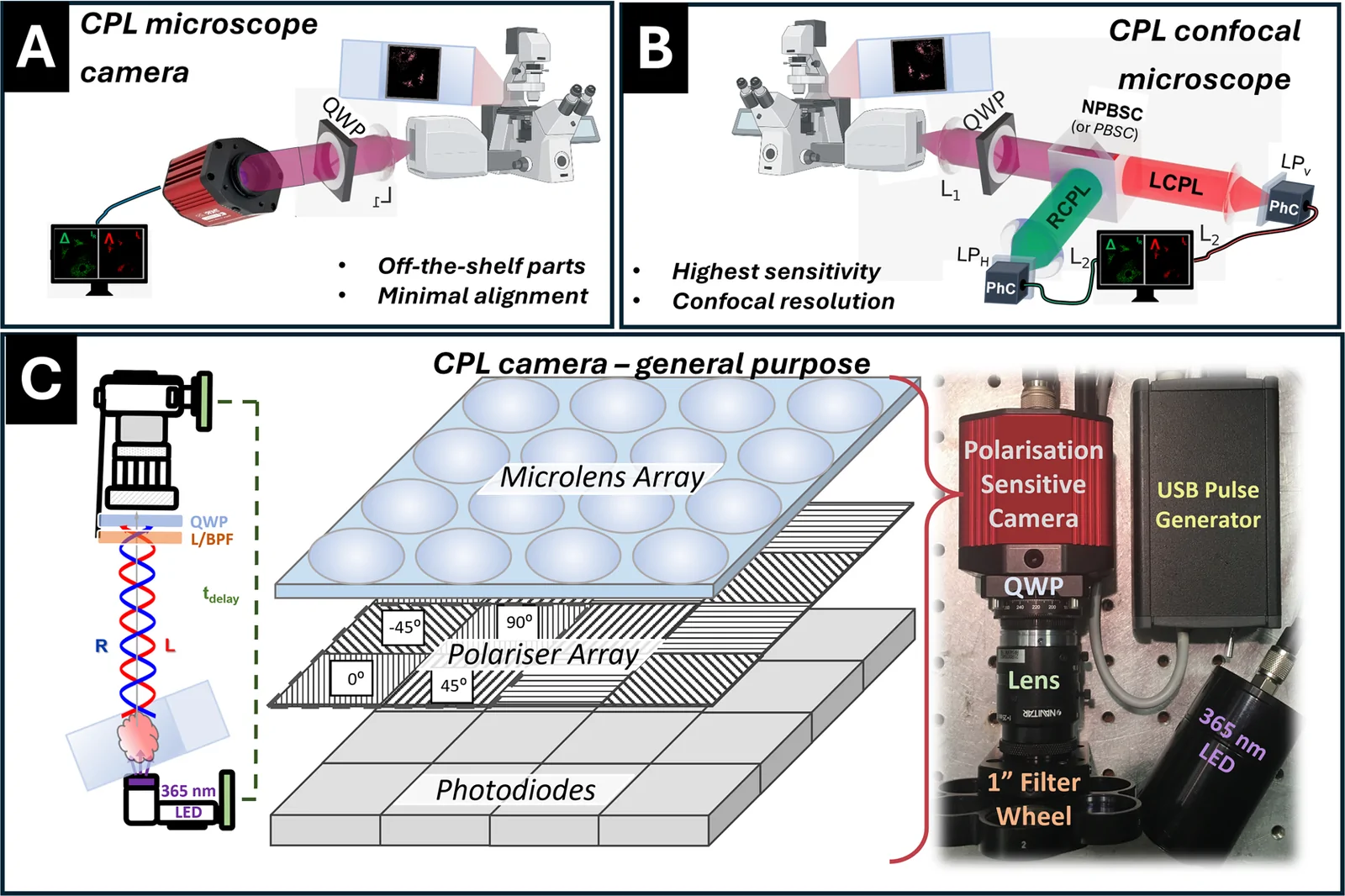
CPL EDCC detection with microscopes and cameras. (A) EDCC CPL camera microscope camera set-up incorporating a polarisation sensitive camera. QWP = quarter-wave plate. (B) CPL EDCC confocal microscope first reported by Stachelek *et al.* [38] and used in subsequent studies [39]. NPBSC = non-polarizing beam splitter cube; LP = linear polarizer, horizontal (h) and vertical (v); PhC = photon counter. (C) A compact CPL EDCC camera system reported by De Rosa *et al.* [42], incorporating a commercial polarization-sensitive camera. Sub-figures reproduced under a Creative Commons Attribution 4.0 International License.

Given the trend towards simplified CPL detection, it is reasonable to expect that CPL detection modalities will be adapted to plate-reader format in future years. CPL plate readers would assist with high-throughput chirality screening, particularly of CPL-active probes and how they interact with biological samples.

Regarding detectability of CPL signals, the degree of CPL emission from an emissive system is quantified by the emission dissymmetry factor, $g_{em}=\frac{2(I_{LCPL}\;-\;I_{RCPL})}{\left(I_{LCPL}\;+\;I_{RCPL}\right)}\;$, where *I*_LCPL_ and *I*_RCPL_ are the intensity of left- and right-handed CPL signals at a given wavelength, respectively. *g*_em_ =   + 2 corresponds to complete L-CPL emission and *g*_em_ =   − 2 corresponds to complete R-CPL emission. Yet, the key metric for straightforward detection of a CPL-active complex is circularly polarized brightness (CPB). For a material emitting light at an arbitrary wavelength, CPB is defined as $CPB\;=\;\varepsilon \;\Phi \frac{g_{em}}{2}$; where ε is the extinction coefficient of the material and Φ is the quantum yield [40]. High CPB values indicate the compound is capable of emitting large quantities of one-handed (monosign) circularly polarized photons [87]. CPB can also be used to compare the performance of different CPL-active materials, where the maximal observed value of the *g*_em_ is used. CPB can also be calculated for individual transitions; (CPB_*i*_) using branching factor (β_*i*_) which is the ratio between the intensities of the emission band of interest (*I_i_*) and total emission (*I*_Total_) [87]. CPB is particularly useful for applications where a specific wavelength range of the detected light can be selected, for instance, by a band pass filter [42]. In that case, the average *g*_em_ for the selected wavelength region is used to calculate CPB_*i*_*.* Therefore, emission manifolds with a single CPL sign are desired to maximize CPB_*i*_ by avoiding cancellation of circular polarization. Although relatively high CPB values have been reported for some organic materials—such as cyclophanes, BODIPYs, helicenes, pyrenes, ‘chirally adaptive’ triptycenes [88] and some transition metal complexes—the highest CPB values are typically observed in lanthanide(III) coordination complexes, particularly those of europium(III) and terbium(III), with their CPB values being an order of magnitude higher than the next brightest class of compounds [73,88–107]. This is owing to the favourable photophysical characteristics of lanthanide complexes: magnetic dipole-allowed transitions, long-lived excited states and narrow emission bands that arise from minimal perturbation of the emitting states by the surrounding environment [108,109]. These features make lanthanide complexes uniquely suited for applications where high CPL contrast and long-lasting emission are desirable. The emergence of circularly polarized emission as a means of encoding chiral molecular fingerprints adds a new dimension to both molecular diagnostics and device engineering. CPL-active materials have demonstrated utility across a growing number of fields, including live-cell chiral imaging [39,110,111]. Enantioselective sensing [112] circularly polarized organic light-emitting devices [113–119] and bright, high CPB security inks [120]. Recent studies have shown that modest CPB of approximately 4 M^–1^ cm^–1^ is sufficient to discriminate between CPL-active fluorescent enantiomers inside living cells using CPL/EDCC microscopy [39]. A comprehensive catalogue of the CPB values of various complexes is provided in Arrico *et al.* [40]. However, CPB values of supramolecular chiral systems are not yet widely studied or catalogued; this is a prominent gap in the literature.

## 3. Consideration of other chiral analytical techniques

To the best of our knowledge, at the time of writing, only CPL-based technologies (§2) could plausibly serve as a clear-cut imaging assay for mirror life, with the particular hardware advantages of handheld or small laboratory footprints (camera and microscopes, respectively), and the development of robust optical technologies that make remote deployment plausible (e.g. via robotic probes).

There are however, a gamut of chiroptical spectroscopy techniques, which could foreseeably be used to analyse biological chirality in relation tomirror life [121]. Examples include circular dichroism (CD) spectroscopy, microplate-format CD [122], CD microscopy; 2nd harmonic circular dichroism (2HCD) microscopy [123]; optical rotation/rotary dispersion; Rayleigh Optical Activity (RayOA) [124] and Raman Optical Activity (ROA)[125]. A key challenge for these techniques is meeting the spatial resolution required for identification of mirror life. For example, there are various CD-based chiroptical assays which have been demonstrated spectroscopically but not via spatial imaging, for example, assays based uponchiral nanoscale molecular assemblies for detection of ferrocene or chiral nanoparticle assemblies for detection of silver ions [126,127]. The full gamut of such CD-based assays would require an extensive review on their own merit. There are also recent cutting-edge developments with regard to circularly polarized light detection technologies, such as the trap-assisted circularly polarized organic photodetectors currently realized in 4096-pixel array format, which may also prove useful for chirality imaging assays when developed further [128]. We encourage the wider scientific community to discuss if and how these techniques could be used for mirror life discrimination.

Regarding other analytical techniques. Electron microscopy can be used to directly image the chirality of biologicalstructures [8], but electron microscopes are bulky, expensive, non-mobile pieces of equipment, requiring specialist laboratories and skilled operators; therefore, any suspected mirror life sample would have to be contained and transported for analysis. Likewise, chiral mass spectroscopy (MS) analysis has been developed, offering high-sensitivity chiral analysis [129–131]. However, the required MS instruments, like electron microscopes, are large, expensive and sensitive scientific apparatus, poorly suited to rapid *in situ* screening. Similarly, chiral high-pressure liquid chromatography is, by its nature, time-consuming and typically does not allow for screening of many samples. Therefore, these other analytical techniques—as currently used—seem to be fairly impractical for *in situ* mirror life.

We encourage other researchers who are experts in these techniques to consider how methods and equipment could be adapted and used for screening against mirror life, and where complementary analysis may be practicable.

## 4. Conclusions

In this perspective, we have set out the first known assay principle to discriminate against mirror life. Our approach is based upon CPL emission from fluorescent chiral and achiral subcellular probes (e.g. organometallic lanthanide complexes, BODIPY dyes, achiral DNA stains, etc.). When localized inside cells, enantioselective binding of these probes can confirm chirality of organelles and molecules, thereby providing a signature of mirror life. Read-out could be via rapid CPL spectroscopy (for probe development), CPL EDCC confocal microscopy (for highest quality subcellular analysis) and CPL EDCC cameras (for *in situ* site inspection or machine vision/remote robotic probes). So far, these read-out technologies have been demonstrated at laboratory scale and could be developed further for specific applications. Indeed, the fundamental optics behind these technologies are analogous to low-cost three-dimensional cinema glasses, and so we believe these technologies are amenable to more widespread production and application.

We have identified several technical considerations and recommendations for future work. Firstly, a wide variety of achiral and chiral CPL-generating probes could be used for mirror life screening—their application to subcellular labelling should be considered and reported in a range of conventional chiral biological specimens. Secondly, we note the need to quantify the CPB metric of achiral fluorophores generating CPL via supramolecular chirality, in order to develop achiral mirror life probes, e.g. for discrimination between conventional and mirror DNA. Thirdly, we note that a rigorously tested multi-reporter panel approach would be required to avoid false positives and ensure robust screening against potential mirror life biohazards. We believe that such reporter panels could be tested on conventional terrestrial life with no mirror life risk. We note that close coordination between biologists and chemists will be required to provide a library of fluorescent CPL-active biological probes that can robustly identify mirror life if it is encountered.

The CPL method could provide a first-line assay foridentifying extant mirror life biohazards, while also offering a foundation for bio-surveillance efforts that would likely be necessary for practical safeguarding against artificial mirror life.

## Data Availability

This article has no additional data.
